# Writhing Movement Detection in Newborns on the Second and Third Day of Life Using Pose-Based Feature Machine Learning Classification

**DOI:** 10.3390/s20215986

**Published:** 2020-10-22

**Authors:** Iwona Doroniewicz, Daniel J. Ledwoń, Alicja Affanasowicz, Katarzyna Kieszczyńska, Dominika Latos, Małgorzata Matyja, Andrzej W. Mitas, Andrzej Myśliwiec

**Affiliations:** 1 Institute of Physiotherapy and Health Science, Academy of Physical Education in Katowice, 40-065 Katowice, Poland; i.doroniewicz@awf.katowice.pl (I.D.); alicjaaffa@gmail.com (A.A.); kieszczynskakasia@gmail.com (K.K.); latoska@poczta.fm (D.L.); m.matyja@awf.katowice.pl (M.M.); a.mysliwiec@awf.katowice.pl (A.M.); 2 Faculty of Biomedical Engineering, Silesian University of Technology, 41-800 Zabrze, Poland; andrzej.mitas@polsl.pl

**Keywords:** infant, feature extraction, classification, machine learning, general movement assessment, physiotherapy, diagnosis

## Abstract

Observation of neuromotor development at an early stage of an infant’s life allows for early diagnosis of deficits and the beginning of the therapeutic process. General movement assessment is a method of spontaneous movement observation, which is the foundation for contemporary attempts at objectification and computer-aided diagnosis based on video recordings’ analysis. The present study attempts to automatically detect writhing movements, one of the normal general movement categories presented by newborns in the first weeks of life. A set of 31 recordings of newborns on the second and third day of life was divided by five experts into videos containing writhing movements (with occurrence time) and poor repertoire, characterized by a lower quality of movement in relation to the norm. Novel, objective pose-based features describing the scope, nature, and location of each limb’s movement are proposed. Three machine learning algorithms are evaluated in writhing movements’ detection in leave-one-out cross-validation for different feature extraction time windows and overlapping time. The experimental results make it possible to indicate the optimal parameters for which 80% accuracy was achieved. Based on automatically detected writhing movement percent in the video, infant movements are classified as writhing movements or poor repertoire with an area under the ROC (receiver operating characteristics) curve of 0.83.

## 1. Introduction

Neuromotor development is a process aimed at achieving full mobility and independence by an infant. It runs in a planned order, so that in healthy developing infants, the sequence of movements is the same and repetitive. At the same time, it has a cascade character, consisting of the fact that after reaching one motor activity, another one follows, while the dynamics of development is not uniform and is based on the so-called development leaps. However, child development is a continuous and coherent process in which the skills achieved in individual development spheres are correlated [1]. Many factors can disrupt this process. It is therefore important that early and comprehensive diagnostics detect them as early as possible to receive appropriate treatment and therapy [2].

The detection of abnormalities in an infant’s psychomotor development, which means the earliest diagnosis possible after birth, is a key stage in ensuring the best quality of life and functioning that can be achieved by the child. For this potential to be fully utilized in practice, it is necessary to use effective and precise tools to verify the neuromotor development at an early stage of the child’s life. This verification is essential for the diagnosis of cerebral palsy (CP) and other developmental disorders. Early diagnosis allows starting the therapeutic process, thus reducing the likelihood of sensory disorders, coordination problems, and postural disorders in children [1].

Depending on the age of the child and the aim of the physiotherapeutic examination, child development is evaluated using both methods based on subjective perception supported by the knowledge and experience of the physiotherapist or the physician and the objective methods using measurement tools. Regardless of the feature being measured, each test should be characterized by the highest possible parameters of effectiveness. Among subjective methods, the Prechtl general movement assessment (GMA) is characterized by high sensitivity and specificity regarding CP [3]. GMA takes into account the full complexity of the child’s nervous system. It is a safe method that can be used in a hospital, even in intensive care settings [4].

In GMA, the evaluation of infant development is based on the analysis of previously recorded videos and the assessment of the quality of general movements. This method is now widely used in the diagnosis of infants to detect the dysfunction of the central nervous system in early life [5].

General movements are a component of the child’s spontaneous activity from nine to 12 weeks of fetal life. They are characterized by a varied sequence of movements of the upper and lower limbs and body trunk. Their intensity, strength, and velocity increase and decrease and have a gradual beginning and end. Mostly, these are complex sequences of extension and flexion of the limbs with superimposed rotations and frequent small changes of direction. These components make movement smooth and elegant and give the impression of complexity and variability [6].

In the period from birth at term age up to 6–9 weeks of postmenstrual age, general movements are called writhing movements. After this period, in the proper development of the infant, writhing movements are replaced by fidgety movements. These are general, circular movements of small amplitude and variable acceleration throughout the body. They are present continuously in an awake infant [7].

Normal writhing movements are characterized by the amplitude of movement ranging from low to moderate and speed ranging from low to moderate. Quick and extensive extension movements may occur sporadically, especially in the lower limbs. Usually, such movements have the form of an ellipse, which creates the impression of a writhing motion. If the general movements lose their complexity and diversity, they are divided into poor repertoire (PR), cramped-synchronized, and chaotic general movements [7].

Poor repertoire movements are observed when the movement sequences are monotonous, whereas the movements of individual body parts do not show complexity [8]. Poor repertoire movements are often found in infants, and therefore, their predictive value is considered in the literature as low [7,9]. Furthermore, in a 10 day observation of newborns, de Vries and Bos concluded that if at least one normal writhing movement is observed in a newborn, the chances for normal development are high (94%) [10].

Cramped-synchronized general movements are characterized by intermittent movements that lack smoothness, with the limb and torso muscles contracting and relaxing simultaneously. If cramped-synchronized movements are observed for several weeks, they have a high predictive value for CP [8,11]. Abnormal cramped-synchronized general movements are sometimes preceded by chaotic movements, which lack smoothness, are characterized by high amplitude, and are chaotic [7,8].

Due to the few objective methods available to examine children at this age, it is important to analyze and compare the results obtained qualitatively to yield more complete and reliable data and predict further psychomotor development. There are many more subjective tools or scales than objective methods. There is a clear lack of equipment that can objectively monitor child development from birth to the age of one year. The measure of objectivity is the comparability of the results obtained by different evaluators [12]. In this respect, the scales rely on the therapist’s perception, which is limited for objective reasons. Further development of research tools must consequently involve the widest possible use of innovative technological solutions, including IT tools, which can significantly support the diagnosis in terms of the quantity of data and the possibility of calculations, measurements, and forecasts [13].

Computer-aided diagnosis of newborns is an area that has been intensively developed in recent years [14]. The starting point for experiments is most often the diagnostics based on the GMA as it does not require a direct interaction between the person conducting the examination and the child. Information about spontaneous motor activity of a child has been collected by researchers in various ways, e.g., using wearable sensors [15] such as accelerometric [16,17], IMU [18,19], and electromagnetic sensors [20]. Although the wearable sensors allow for obtaining good results in the analysis of adults movement [21], the question of the effect of body-mounted elements and their wiring on the child’s spontaneous movements remains debatable. Due to the increasing capabilities of computer image processing, various solutions based on video analysis [22] and using optical systems for depth image acquisition (RGB-D) [23] are currently being proposed. Compared to sensor data, the use of video-based data reduces the impact of the measurement on the child, offers more opportunities for practical use, and ensures better results [24]. In individual studies, authors have attempted to identify irregularities, mostly by reducing the problem to the classification of the recording into the normal and abnormal groups. The proposed solutions can be divided according to the method of the extraction of features describing the child’s movement into those based on features extracted directly from the recording (optical flow, background subtraction) [25,26] and pose-based features, in which the extraction of features is preceded by the process of locating individual body segments [27,28]. Currently, the extraction capabilities of pose-based features have improved due to the availability of ready-made human pose estimation libraries such as OpenPose [29], whose accuracy in the case of infant movement analysis has been confirmed in various studies [28,30].

In the present study, the approach based on video recordings of newborns is shown. These data were the necessary basis for the diagnostics using the GMA. Due to the very low age of the examined newborns, the data acquisition process had to be performed in the neonatal unit. The measurement stand has been prepared in such a way that it is possible to easily carry out the recording procedure by the hospital staff. The use of additional wearable sensors would extend the length of additional procedures for installing sensors on the child’s limbs and running data acquisition software. It would also be necessary to monitor the condition of the sensors and ensure that they are always ready to work, e.g., by keeping the battery charge high. The use of active sensors in this case could also raise parents’ doubts about the infant’s safety, which would make it more difficult to obtain enough data.

### Aim of the Study

The aim of the present study is to use machine learning methods to detect writhing movements on video recordings of children on the second and third day after birth. The authors attempted to determine the indices of spontaneous motor activity of a child that allow for its quantitative description. The proposed parameters must be normalized in such a way that their values do not depend on the parameters of the camera, the child’s body dimensions, and its position relative to the camera. Another criterion that must be met is the possibility of the easy interpretation and reference to the observation made by the diagnostician. Based on the parameter values obtained for different time intervals, we attempted to use popular machine learning methods for the detection of writhing movements.

## 2. Materials and Methods

The study was approved by the Biomedical Research Ethics Committee (No. 5/2018) and in accordance with the Declaration of Helsinki. The study was approved by the Bioethics Committee of Research of the Jerzy Kukuczka Academy of Physical Education in Katowice.

### 2.1. Dataset

The study used a database of 125 recordings of newborns collected in cooperation with the Neonatal Unit at Public Hospital in Piekary Śląskie, Poland. The measurement stand consisted of a 1 m × 1 m tabletop and a frame with a camera mounted 1 m above the tabletop surface. The stand was equipped with a HDR-AS200V video camera (Sony Corporation, Tokyo, Japan), enabling recording at a spatial resolution of 1920×1080 px at a 60 fps sampling rate.

The study involved children on the 2nd or 3rd day after birth. The inclusion criteria in the experiment were the uneventful course of pregnancy and the children who had been born full-term (38–42 weeks) with an Apgar score of 8–10. After obtaining the parent’s written consent to participate in the study, the hospital staff started the recording procedure. The child in the hospital bed was placed on the tabletop of the stand, and then, the camera was activated. During the examination, the child was under the supervision of the researcher. If a child cried, the recording was interrupted and, if possible, repeated later. The recommended time for correct recording was at least 10 min.

**Figure 1:**
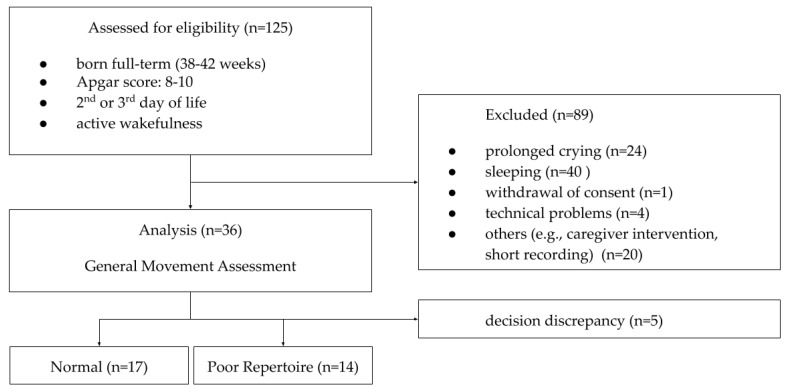
Flow diagram containing criteria for excluding recordings from further analysis and the division resulting from the application of GMA.

The collected recordings were evaluated by an expert for compliance with the active wakefulness states 3 (eyes open, no movements) and 4 (eyes open, gross movements), according to Prechtl’s classification of states [31]. The videos that showed longer fragments of crying or sleeping of a child were removed from further analysis. In some cases, the caregiver’s intervention to calm the child by rocking, stroking, feeding, or giving a pacifier was observed during the recording, which also excluded the recording from further analysis. Meeting the recommended criteria for effective GMA proved to be extremely difficult in the test conditions. Of all 125 recordings, thirty-six were included in further analysis. These recordings were then evaluated by 5 experts in the field of diagnostics using the GMA and divided into groups with normal WM and PR movements. In 26 cases, the experts were in full agreement on the decisions made (WM: 14, PR: 12). In the case of divergences in classification, the final decision was made jointly. Eventually, the dataset was divided into 17 recordings containing normal WM and 14 containing PR movements. Differences between the experts in the other 5 cases determined the exclusion of these recordings from the classification stage (Figure 1).

**Figure 2:**
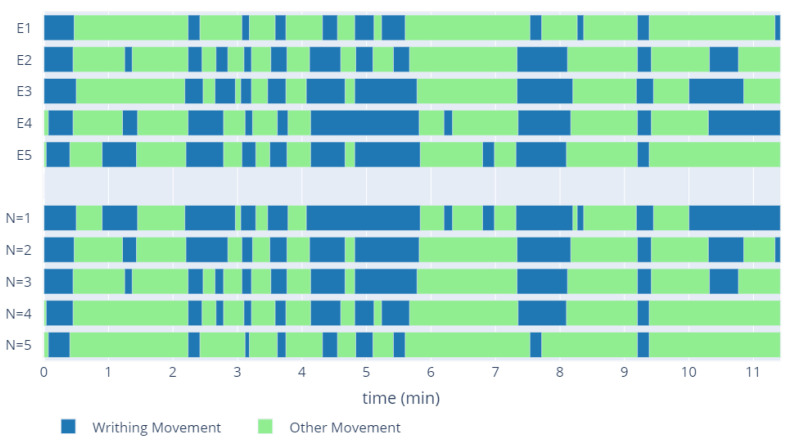
Examples of indications of WM fragments by different experts (E1–E5) for one recording. The intervals denoted N = 1–5 present the ground truth depending on the number of experts for whom the common part was chosen.

In the recordings from the normal WM group, each expert independently identified the parts in which the child presented the writhing movement. Due to the discrepancies between the experts, training data preparation required the determination of one set of WM fragments for each recording, in the form of a common part for N experts (Figure 2). Depending on the number of experts for whom the common part was selected, the number and length of the WM fragments changed. The WM time in the training set was longer for low N values. Increasing the number of experts shortened it, while increasing the confidence of the classification. Selection of the appropriate N parameter was one of the evaluation elements of the proposed models.

### 2.2. Video and Pose Preprocessing

The study used the pre-processing methodology proposed by the authors in the previous pilot study on computer-aided infant movement analysis. In order to reduce the impact of the camera’s optical system, the recordings were subjected to a procedure of distortion removal. The camera parameters were determined by calibration using the checkerboard pattern. Then, from the whole recording, the area containing the hospital bed with a newborn was manually indicated [13].

Pose estimation was performed using the OpenPose library [29]. The processing yielded 25 landmarks indicating the location of individual joints and characteristic points. Results with low detection confidence were replaced by previous point locations. The observation of the detected points revealed the occurrence of vibrations in the form of high-frequency noise resulting from the inaccuracy of the detection of a given point on subsequent frames of the video. Removal of these disturbances without affecting the overall movement trajectory was performed using the Savitzky–Golay filter, which enables smoothing the signal without significant distortion [32]. In order to reduce the impact of the arrangement and the changes in the body position on the results, the coordinates of the points from each frame were translated so that the neck point did not change its position during the recording. Then, the entire body was rotated in such a way that the axis of the child’s body was parallel to the axis of the image. A similar approach for the pre-processing of the OpenPose results in the analysis of infant recordings was previously proposed by various research teams [13,28,30].

### 2.3. Feature Extraction

In previous studies, we verified the possibilities of describing the scope, nature, and location of limb movements of children in the period of fidgety movements using the parameters of the ellipse circumscribed on the movement trajectory [13]. A methodology for normalization of the proposed parameters based on limb length was also developed, making it possible to use them for the objective description of limb movement. This allowed for the observation of their variability depending on the head position during a few minutes of observation [33]. In the present experiment, the previously proposed features of the FMA (factor of movement’s area), FMS (factor of movement’s shape), and CMA (center of movement’s area) were determined using shorter analysis time: from 5 to 30 s, and different times of overlapping of the subsequent analysis frames.

**Figure 3:**
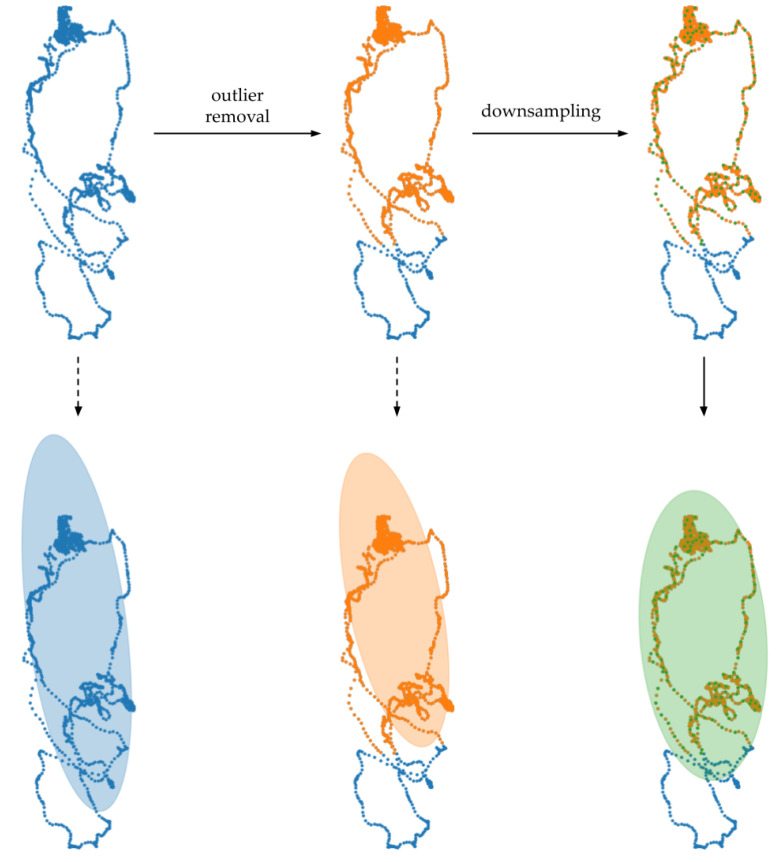
Effect of individual trajectory processing steps on the resulting shape of the ellipse. Omitting pre-processing leads to the inclusion of an area not covered by the trajectory in the ellipse. The removal of outliers reduces the effect of single parts of trajectories deviating from the general course of motion, but the result is still affected by areas of density resulting from the low amplitude of motion. Downsampling in the form of removing adjacent points reduces the excess area of the ellipse and leads to changes in its orientation due to the areas of density.

Using the trajectories of the distal parts of the limbs, a set of features was determined based on the parameters of the ellipse circumscribed on each trajectory. In the first stage of processing, the effect of single deviations in the trajectory was reduced. For all locations of a point vs. time, its Euclidean distance from the trajectory centroid determined as a mean value from each coordinate was calculated. Then, based on the interquartile range rule, points with a distance greater than $Q_{3}+1.5IQR$ were deleted. The next step was downsampling used to reduce the accumulation of trajectory points at the moments of limb stops. The ellipse parameters were determined based on the eigenvectors and eigenvalues of the covariance matrix of other trajectory coordinates. The pre-processing and its effect on the resulting shape and position of the ellipse are shown in Figure 3.

The values of the determined features expressed in the domain of the analyzed image depend on the camera parameters and the length of the child’s body and limbs. The individual features were normalized to obtain an interpretable value in relation to the actual movement parameters, regardless of the factors indicated. The independence from the proportions of the child’s body also allows increasing the reliability of the comparison of the values obtained between the individual children. The procedure for measuring the length of a child’s limbs in the domain of the image was performed automatically based on the location of the joints. For each frame, the Euclidean distance was determined between three consecutive points: shoulder, elbow, and wrist for upper limbs and hip, knee, and ankle for lower limbs. Next, the maximum value was selected, corresponding to the situation where the limb was placed parallel to the image plane.

**Figure 4:**
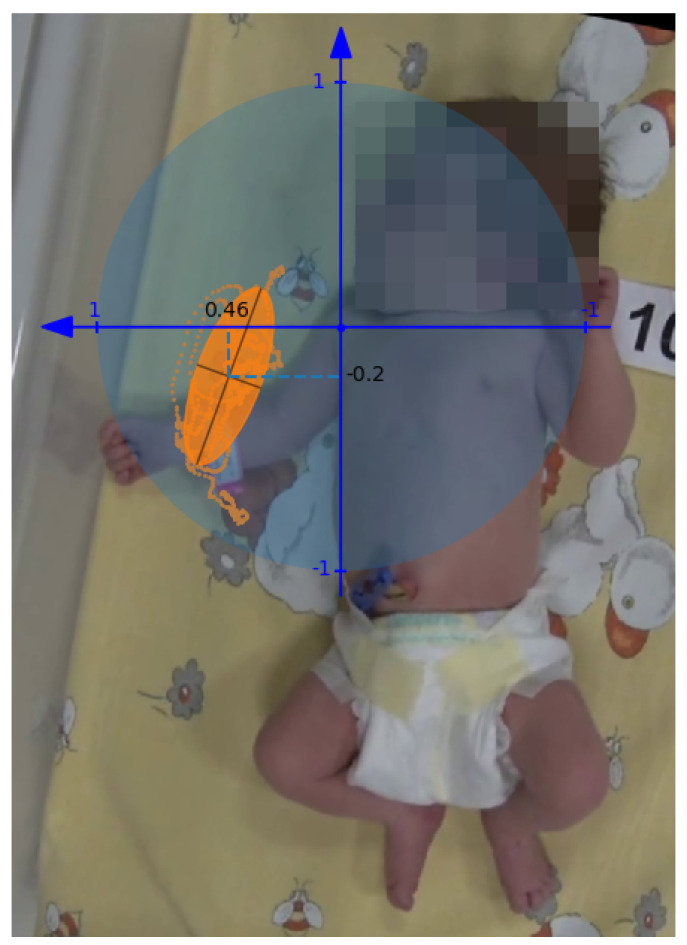
Visualization of the coordinate system associated with the right shoulder used to normalize the parameters of the ellipse circumscribed on the trajectory of the right wrist from a 30 s observation. The unit of the system is the length of the analyzed limb, whereas the sense of the horizontal axis is oriented towards the body axis. The blue circle shows the possible range of motion used to normalize the value of the area of the ellipse circumscribed on the trajectory (orange). Minor and major axes are marked inside the ellipse.

FMA was obtained by dividing the area of the ellipse expressed in pixels by the area of the circle with a radius corresponding to the limb length. Its value can be interpreted as the ratio of the motion performed to the maximum possible range of motion. The FMS parameter corresponds to the aspect ratio of the ellipse determined by dividing the length of the minor axis by the length of the major axis. The position of the center of the ellipse was related to the coordinate system associated with the proximal joint of the analyzed limb: shoulder joint for the upper limb and hip joint for the lower limb. The unit of the system is determined by the length of the limb; the sense of the vertical axis is always oriented towards the head, whereas the sense of the horizontal axis depends on the analyzed side and points to the distal direction (from the body axis) (Figure 4). The FMA, FMS, and horizontal and vertical components of CMA give 4 values describing the movement of each limb. In the classification process, these 4 values for 4 limbs were concatenated into a vector with 16 features characterizing spontaneous movement of the infant.

### 2.4. Classification

The problem of detecting the instants in the recording when the newborn performed writhing movements is considered a binary classification problem. Training data were prepared based on the time intervals of writhing movements defined by 5 experts in the individual videos. Feature extraction was performed for different lengths of the analysis window. The resulting feature vector containing FMA, FMS, and CMA for each limb contained 16 values of features. Each analyzed window was marked as WM (writhing movement) or OM (other movement) depending on the proportion of frames marked as WM by experts.

A leave-one-out cross-validation was performed to exclude one recording from the training set. Then, the training data from the other 30 videos were used to train the machine learning algorithm. The result of the model for the test case allowed for the determination of the evaluation metrics such as accuracy, sensitivity, specificity, and F1 score [34]. Three machine learning algorithms were selected for classification: support vector machine (SVM) with the radial basis function (RBF) kernel, random forests (RF), and a classifier based on linear discriminant analysis (LDA). The testing procedure was conducted for different time frames and overlapping times. In overlapping moments, the final classification was based on the more frequently indicated class, and in the case of equal division, the fragment was classified as other movements.

## 3. Results

The first step of the analysis of fragments containing WM was to compare experts’ agreement on the time of their occurrence on recordings. For this purpose, for each pair of results, the accuracy and F1 score parameters were calculated from all 17 recordings containing WM (Table 1).

**Figure 5:**
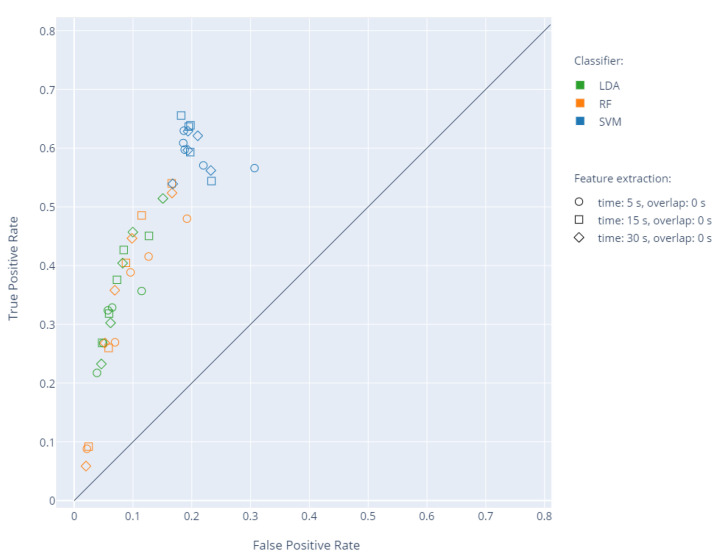
The individual results of cross-validation in the true positive rate (TPR) to false positive rate (FPR) space depending on the time window and the selection of a training set with different N values. The change in parameter N is represented by five identical markers for each pair of classifier and feature extraction window.

The mean accuracy between the experts was 81.89, with an average F1 score of 79.44. For the whole set of 31 WM and PR recordings, feature extraction was performed for 5, 15, and 30 s non-overlapping time windows. Next, leave-one-out cross-validation was carried out five times, changing the N parameter affecting the training dataset. Based on the results, the sensitivity and specificity were determined for a given N in relation to the ground truth. The results showing the relationship between the true positive rate (TPR) and false positive rate (FPR) for individual classifiers are presented in Figure 5.

**Figure 6:**
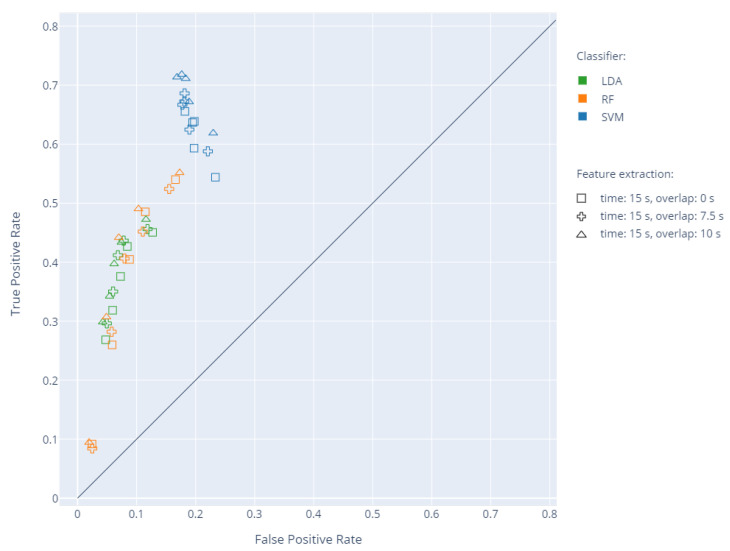
The individual results of cross-validation in the TPR to FPR space depending on the overlapping time for the analysis time of 15 s and the selection of a training set with different N values. The change in parameter N is represented by five identical markers for each pair of classifier and feature extraction window.

Subsequent analyses were extended by overlapping time, which was changed to have 12 or 23 of the window length. Figure 6 presents the results of individual classifiers for a time window of 15 s with overlapping of 0, 7.5, and 10 s, respectively. The increase in N for LDA and RF led to an increase in sensitivity while reducing specificity. For SVM, the increase in N did not negatively affect specificity, allowing reaching high sensitivity values. The use of the overlapping time of 10 s and N = 3 improved the sensitivity of each classifier, with similar or higher specificity values. The exact values of sensitivity and specificity and the corresponding accuracy and F1 score are presented in Table 2.

A comparison of the classification results with the division indicated by each expert was performed by calculating the accuracy and F1 score between the results for the entire set of videos containing WM (Table 3). These results can be compared with Table 1 using the classifier as the next expert. The last column shows the average value of each parameter.

**Figure 7:**
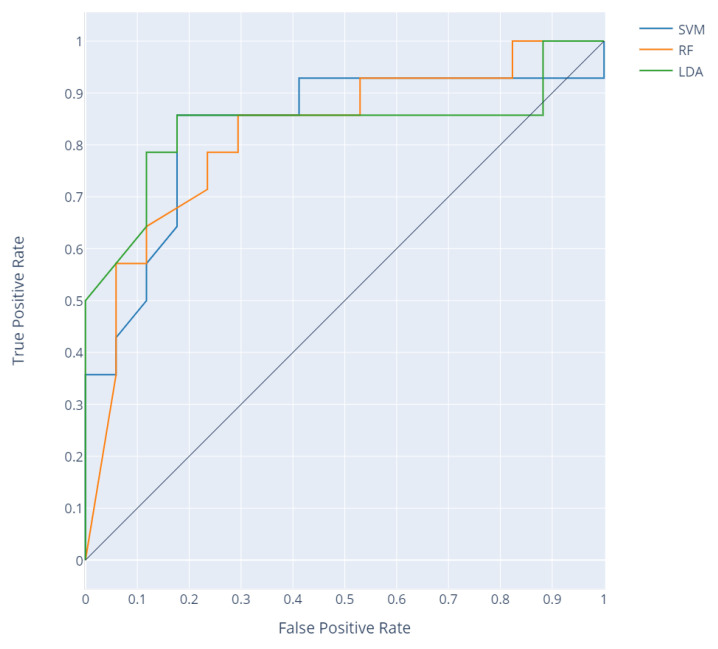
Receiver operating characteristic (ROC) curve for the division of the entire set of recordings into writhing movements and poor repertoire movements based on the percentage threshold of WM in predictions for each classifier.

Accurate detection of normal writhing movements and their differentiation from poor repertoire movements should allow classifying recordings according to the division made by experts. The criterion for group division was the percentage of detected WM movements in a given recording. The receiver operating characteristics (ROC) curve was obtained by increasing the threshold value and determining the sensitivity and specificity for each classifier (Figure 7). The resulting area under the ROC curve (AUC) values are shown in Table 4.

## 4. Discussion

This study attempted to automatically detect normal writhing movements based on the proposed set of original features using pose estimation of video recordings. In the study, the experts analyzed the recordings of 125 healthy newborns on the second and third day of life in order to select a group meeting the criteria for inclusion in the classifier preparation stage. Eventually, at least 10 min recordings of active waking were obtained in the group of 36 newborn babies included in the study. The restrictive criteria adopted at this stage of the examinations were aimed at obtaining a reliable set of data allowing for the proper validation of the proposed automated detection methodology. Fragments from rejected recordings may be included in future research to extend the methodology proposed in the study.

The classification of recordings into normal writhing movements and poor repertoire movements by five experts suggests the high reliability of the division obtained. This is particularly important due to the age of newborns in the study group. The application of general movement assessment in the first week of life is a big challenge and is characterized by a low diagnostic value in the context of the future prevalence of abnormalities during neurological development [5]. A large percentage of newborns develop normal writhing movements after about seven days of life, previously showing poor repertoire movements [9]. This is confirmed by the division into recordings containing WM and PR in the analyzed group of healthy newborns.

Several tests were performed, involving leave-one-out cross-validation of three classifiers: SVM, RF, and LDA, using the proposed numerical indicators (FMA, FMS, and CMA) describing the range, nature, and location of the movements of individual limbs. The change of the window length and overlapping time and the training set by modifying the N parameter (Figure 4) influenced the obtained values of sensitivity and specificity (Figure 5 and Figure 6). Based on the analyses, an optimal set of parameters was selected: window length of 15 s with an overlapping time of 10 s and N = 3 due to a good ratio of sensitivity and specificity for the SVM classifier (Table 2). For the proposed features, low sensitivity values were obtained using RF and LDA. The analysis of individual cases showed that the decrease in sensitivity is mainly due to problems with the detection of the entire WM fragments in specific recordings by all three classifiers. The SVM is better at detecting longer and consistent fragments of WM, whereas RF and LDA find a few smaller fragments, while omitting a greater number of whole WM fragments. The indication N = 3 confirms the thesis that more than one expert should be consulted. The increase in N between one and three in each case resulted in a significant increase in sensitivity and, in the case of SVM, also an increase in specificity. The indication of the exact beginning and end of the movement was a problematic task for the experts because in practice, this information is not important for making an accurate diagnosis. The choice of a common fragment for a greater number of experts eliminated the effect of uncertain redundant fragments on the learning process. The use of overlap in WM detection is justified by the way physiotherapists work, since in practice, when they make a decision concerning a given instant of a recording, they take into account the state before and after the fragment.

The comparison of WM indications as an effect of individual classifiers was compared with the indications of individual experts taking into account only recordings from the group containing WM (Table 3). The highest values of the accuracy and F1 score were obtained for the SVM. These values are lower than the parameters comparing indications between experts (Table 1). This comparison allows for verification of the capability of the classifier to distinguish normal writhing movements from other movements (or rest) that are not poor repertoire movements. The lower values are mainly due to the SVM’s failure to indicate long WM fragments in several recordings.

The use of the proposed methodology to classify entire recordings into those containing normal WM and those containing poor repertoire was verified by drawing the ROC for a variable percentage threshold of WM proportion in the recording (Figure 7). This aimed to check the possibility of automated WM classification in the screening indication of the recordings containing poor repertoire movements (positive class). AUC values above 0.8 were obtained for all three classifiers. The parameter was affected in this case by the high specificity of each classifier. In the best case for the SVM classifier, twenty-six recordings were classified correctly, with three false positives and two false negatives. The AUC value of 0.83 for this classifier can be compared with the classification made by experts. In five of 36 cases, the decision on proper classification was not obtained. The remaining resolved 31 expert classifications gave 86% of certain decisions in the division of recordings into those containing WM and those containing PR.

To the best of the authors’ knowledge, this study is the first attempt to indicate fragments of WM on recordings of newborns in the first days of life. In the cases considered so far, the prevalence of fidgety movements in newborns from the age of two months has been considered more frequently, especially in the context of early detection of the risk of cerebral palsy [35]. An approach similar to that presented in this study was used by Ihnen et al. [27], who analyzed cerebral palsy prediction in a large group of recordings of children over nine weeks of age. Their training set was built based on the classification of the entire recordings, and then each 5 s of the recording were classified. The division of recordings was made based on the diagnosis of CP in a child in the next period of life, into CP and non-CP classes. The proposed model was built based on features determined using the trajectory of selected body segments for every five seconds of recording. The classification of the recording was based on the percentage of CP fragments, reaching AUC = 0.87. In a study by McCay et al. [28], an attempt was made to automatically divide short recordings from the MINI-RGBD database [23] into normal and abnormal movements based on pose-based features. The proposed histogram-based features ensured the high accuracy of the automated grouping of 12 recordings using SVM, LDA, and different neural network architectures. The results indicated high accuracy of the classification of spontaneous movements in newborns based on features describing only the movement of limbs using neural networks. The problem of the automated detection of normal writhing movements and poor repertoire movements was addressed in a study by Tsuji et al., which discussed the classification of 30 s fragments of recordings into four types of general movements [26]. Using a log-linearized Gaussian mixture network and features based on the motion image, the authors achieved high accuracy to distinguish between normal and abnormal movements. However, only previously selected fragments of video recordings containing general movements were used in the study, without periods of crying, sleep, or lack of movement, which, compared to the analysis of the whole recording, could have contributed to an increase in accuracy.

The latest publications in the field of computer-aided diagnostics of infants indicate the direction of further development towards solutions based on human pose estimation algorithms [26,27]. The results of the research using this approach show the great potential of the OpenPose library for this type of application [28,30,36].

This study proposed intuitive spatio-temporal pose-based features describing spontaneous child motor activity and confirmed the possibility of their use in the detection of writhing movements in recordings of children on the second and third day of life. Due to the lack of newborns with confirmed neurodevelopmental abnormalities in the analyzed group, the effectiveness of the proposed method in diagnostics support must be confirmed by subsequent studies. Another limitation of the present study is the size of the group, which should be increased, in particular, with high-risk newborns. The proposed features describe infant’s motor activity in only two dimensions, which results in a loss of information that may be important for a quantitative description. In further stages of the development of the presented solution, the authors plan to extend the set of features with parameters describing the change in the position of the body trunk and head. During experts’ observation, this information plays an important role in decision making, so it may positively affect the automatic classification results. It is also planned to extend the range of classification algorithms tested, in particular with different neural network architectures and deep learning approaches. Further research also assumes the use of useful fragments of recordings rejected at this stage. It is also necessary to develop a methodology for the initial indication of moments of movement without crying of the child, which, combined with a greater diversity of data sets, should yield better results.

## Figures and Tables

**Table 1:** Accuracy and F1 score between writhing movement (WM) fragments selected by individual experts pairwise.

	E1	E2	E3	E4	E5
**E1**	-	78.60	80.78	77.44	81.18
**E2**	75.45	-	83.04	78.85	80.93
**E3**	77.54	81.88	-	85.09	86.28
**E4**	73.23	77.08	83.56	-	86.72
**E5**	77.23	78.98	84.60	84.88	-
		Accuracy		F1 score.	

**Table 2:** Classifiers’ assessment metrics obtained by cross-validation across the entire dataset containing WM and PR recordings for the selected analysis time of 15 s, overlapping time of 10 s, and N = 3.

	ACC	SENS	SPEC	F1
SVM	80.23	71.36	83.15	64.13
RF	80.93	44.18	93.02	53.42
DA	80.41	39.70	93.81	50.10

**Table 3:** Accuracy and F1 score between the WM fragments determined by the classifier and individual experts, 15 s, overlapping of 10 s, and N = 3.

	Metric	E1	E2	E3	E4	E5	Mean
SVM	ACC	76.88	74.92	76.39	74.54	77.29	76.00
	F1	70.52	71.25	72.42	69.79	72.54	71.30
RF	ACC	71.29	68.33	69.71	68.55	71.18	69.81
	F1	53.35	55.00	55.93	53.35	56.21	54.77
LDA	ACC	73.00	65.17	68.52	68.35	70.77	69.16
	F1	54.06	48.48	52.29	51.04	53.64	51.90

**Table 4:** Area Under Curve (AUC) for each model in WM/PR classification.

	AUC
SVM	0.83
RF	0.82
LDA	0.84

## Abbreviations

The following abbreviations are used in this manuscript:

WM	writing movement
PR	poor repertoire
OM	other movement
CP	cerebral palsy
FMA	factor of movement’s area
FMS	factor of movement’s shape
CMA	center of movement’s area
GMA	general movement assessment
ROC	receiver operating characteristics
AUC	area under the ROC curve
